# Genomic Analysis of Soybean PP2A-B′′ Family and Its Effects on Drought and Salt Tolerance

**DOI:** 10.3389/fpls.2021.784038

**Published:** 2022-02-02

**Authors:** Yang Xiong, Xu-Hong Fan, Qiang Wang, Zheng-Gong Yin, Xue-Wen Sheng, Jun Chen, Yong-Bin Zhou, Ming Chen, You-Zhi Ma, Jian Ma, Zhao-Shi Xu

**Affiliations:** ^1^College of Agronomy, Jilin Agricultural University, Changchun, China; ^2^Institute of Crop Science, Chinese Academy of Agricultural Sciences (CAAS)/National Key Facility for Crop Gene Resources and Genetic Improvement, Key Laboratory of Biology and Genetic Improvement of Triticeae Crops, Ministry of Agriculture, Beijing, China; ^3^Soybean Research Institute, Jilin Academy of Agricultural Sciences/National Engineering Research Center for Soybean, Changchun, China; ^4^Crop Resources Institute of Heilongjiang Academy of Agricultural Sciences, Harbin, China; ^5^College of Modern Agriculture, Changchun Vocational Institute of Technology, Changchun, China

**Keywords:** protein phosphatase 2A-B′′, genomic analysis, soybean, drought, salt

## Abstract

Abiotic stresses induce the accumulation of reactive oxygen species (ROS) and significantly affect plant growth. Protein phosphatase 2A (PP2A) plays an important role in controlling intracellular and extracellular ROS signals. However, the interaction between PP2A, ROS, and stress tolerance remains largely unclear. In this study, we found that the B′′ subunit of PP2A (PP2A-B′′) can be significantly induced and was analyzed using drought- and salt-induced soybean transcriptome data. Eighty-three soybean *PP2A-B′′* genes were identified from the soybean genome *via* homologous sequence alignment, which was distributed across 20 soybean chromosomes. Among soybean *PP2A-B′′* family genes, 26 *GmPP2A-B′′* members were found to be responsive to drought and salt stresses in soybean transcriptome data. Quantitative PCR (qPCR) analysis demonstrated that *GmPP2A-B′′71* had the highest expression levels under salt and drought stresses. Functional analysis demonstrated that overexpression of *GmPP2A-B′′71* in soybeans can improve plant tolerance to drought and salt stresses; however, the interference of *GmPP2A-B′′71* in soybean increased the sensibility to drought and salt stresses. Further analysis demonstrated that overexpression of *GmPP2A-B′′71* in soybean could enhance the expression levels of stress-responsive genes, particularly genes associated with ROS elimination. These results indicate that PP2A-B′′ can promote plant stress tolerance by regulating the ROS signaling, which will contribute to improving the drought resistance of crops.

## Introduction

Soybean [*Glycine max* (L.) Merr.] is one of the main plant proteins and lipid resources and plays an important role in our national agricultural production; its planting area ranks first among the four oil crops. However, climate change continues to threaten soybean growth, which has resulted in serious losses in yield, especially abiotic stresses such as drought, soil salinization, and high temperatures (Zhu, 2016). During plant evolution, this changing environment causes plants to develop a variety of sophisticated strategies to resist abiotic stresses. Among plant defense systems, the removal of reactive oxygen species (ROS) is important to improving plant tolerance. Research has revealed that abiotic stress can induce a high level of ROS in plants, which can cause plant cell damage (Alam et al., 2021) and reduce plant tolerance. Protein phosphatase 2A (PP2A) is involved in the ROS pathway to accurately regulate cell death and control metabolic changes (Rahikainen et al., 2016).

Protein phosphatase 2A consists of a structural/scaffold subunit (A), a well-conserved catalytic (C) subunit, and a regulatory (B) subunit (Durian et al., 2020). The B subunit interacts with both the C subunit and the A subunit (Xu et al., 2006). The B subunit determines the specificity of the PP2A enzyme and substrate and mediates the role of PP2A in signal transduction (Bheri and Pandey, 2019). Our research demonstrated that the B subunit contains four subfamilies, and which were named B55 (B), B56 (B′, PR61), PR72 (B′′), and PR93 (B′′′), while further analysis shows that these four subfamilies have different primary sequences and tertiary structures (Liu et al., 2014). Motifs analyses demonstrated that the B′′ subunit protein contains EF-hand motifs which are Ca^2+^-binding domain (Leivar et al., 2011; Uhrig et al., 2013), and further study found that the B′′ subunit protein contains at least two or more EF-hand repeat domains and that each motif contains 12 granulated loops flanked on both sides by a 12 residue alpha-helical domain. The binding of calcium ions promotes the conformational change of the EF-hand motif, leading to the activation or inactivation of its target protein activity (Skelton et al., 1994; Ikura, 1996; Yap et al., 1999). This indicates that GmPP2A-B′′ could be involved in the calcium signaling pathway. Calcium ions promote the connection between PP2A-B′′ and the A subunit, while the B′′ family related with dephosphorylation of VirE2-INTERACTING PROTEIN 1 (VIP1) *in vitro*. AtVIP1 protein was transferred from cytoplasm to nucleus under hypotonic conditions and depends on the calcium signaling pathway (Tsugama et al., 2012, 2019). This indicates that PP2A-B′′ plays important roles in maintaining *Arabidopsis* growth under hypo-osmotic conditions (Takeo and Ito, 2017; Tsugama et al., 2018, 2019; Yoon et al., 2021). It was reported that *TaPP2AbB′′-α*, a novel regulatory subunit B, was responsive to NaCl, polyethylene glycol 6000 (PEG6000), cold, and abscisic acid (ABA) at the transcriptional level, and overexpression of *TaPP2AbB′′-α* in transgenic *Arabidopsis* had more lateral roots under mannitol or NaCl treatment. These results indicated that *TaPP2AbB′′-α* involved in various stress responses and promoted lateral root growth under osmotic conditions (Liu et al., 2014). Our research indicates that PP2A participates in multiple signaling pathways.

Protein phosphatase 2A is involved in regulating the multi-node signal network in eukaryotes (Durian et al., 2016). This study demonstrated that PP2A was necessary for the dephosphorylation and activation of brassinazole-resistant 1 (BZR1) in *Arabidopsis*, which mediated regulation of the cascade reaction of brassinolide (BR) signal from cell surface receptor kinase to gene regulation in the nucleus (Tang et al., 2011). Under salt and osmotic stress conditions, ABA binds to PYR/PYL/RCAR (PYLs) and inhibits PP2A activity, which leads to increased PIN-FORMED (PIN) phosphorylation and consequently modulates directional auxin transport, subsequently adapting the root architecture (Li et al., 2020; Yu et al., 2021). Genetic studies in rice (Yu et al., 2005), proteomic, and metabolomic studies (Gabriele Siegll, 1990; Sabine et al., 1993) in plants demonstrated that PP2A controls metabolic changes and cell death elicited by intracellular and extracellular ROS signals, which contributes to the transcriptional and post-translational regulation of pro-oxidant and antioxidant enzymes in plants. However, the relationships between PP2A, ROS, and stress tolerance in soybean remain largely unclear. In this study, we systematically analyzed soybean PP2A-B′′ subunit family members and found that *GmPP2A-B′′71* could improve drought and salt tolerance in transgenic soybeans by activating genes associated with the ROS elimination pathway. Our study provides a theoretical basis on the PP2A-mediated regulation ROS signaling to improve plant stress tolerance in soybean plants.

## Materials and Methods

### Identification of PP2A-B′′ Family Genes in the Soybean Genome

We did a protein BLAST search of the soybean genome in the Phytozome database^1^ to obtain 83 soybean PP2A-B′′ sequences. ExPASy ProtParam^2^ was used to analyze the isoelectric point (p*I*) of each PP2A-B′′ family member amino acid sequence and their molecular weights.

### Chromosomal Distribution, Phylogenetic Analysis, and Multiple Sequence Alignment

Through the Phytozome database, we found that the 83 identified *GmPP2A-B′′* genes were distributed across 20 soybean chromosomes; the distribution map of the 83 *GmPP2A-B′′* family genes on the chromosomes was made by Map Gene 2 Chromosome v2 (MG2C) software.^3^ Eighty-three *GmPP2A-B′′s* and 43 *AtPP2A-B′′s* were used for multiple sequence alignment analysis to construct a phylogenetic tree using the maximum likelihood (ML) method. The phylogenetic tree was constructed using MEGA-X with 1000 Bootstrap replicates.

### Gene Structure, Motif Composition, *Cis*-Acting Elements, and Tissue-Specific Expression Patterns

Gene Structure Display Server 2.0 (GSDS 2.0) software^4^ was used to determine the structures of the 83 *GmPP2A-B′′* genes (Hu et al., 2015). Motif analysis of the 83 genes was performed using Multiple Em for Motif Elicitation (MEME)^5^ software (Bailey et al., 2009). We selected 1500 bp upstream of the ATG start codon of *GmPP2A-B′′12/33/36/46/56/68/69/71/82* to screen for *cis*-acting elements using PlantCARE [PlantCARE, a database of plant promoters and their *cis*-acting regulatory elements (ugent.be)] (Magali Lescot et al., 2002). *Cis*-acting element diagrams were generated using GSDS 2.0. We used Soybase^6^ to identify the expression patterns of the 83 *GmPP2A-B′′* genes in different tissues (Du et al., 2018), and the heat map was made using TBtools. The transcriptome data (PRJNA694374) for the various abiotic stressors was obtained in our previous study (Wang et al., 2021).

### RNA Extraction and Quantitative PCR Analysis

Soybean variety “Williams 82” was used for quantitative PCR (qPCR) analysis. Soybean seeds were sown into flowerpots and after 2 weeks of growth, soybean seedlings were irrigated with 200 mM NaCl and 250 mM mannitol, respectively. A total of 0.1 g of soybean leaf blade tissue was collected at 0, 2, 4, 6, 8, 10, and 12 h after the 200 mM NaCl induced salt stress and 250 mM mannitol induced drought stress treatments, and total RNA was extracted using an RNA extraction kit (ZOMANBIO, ZP405, China). Double stranded cDNA was obtained using a cDNA synthesis kit (TIANGEN, KR118, China) (Le et al., 2011). qPCR reactions were performed on the ABI Prism 7500 real-time PCR system (Applied Biosystems, Foster City, CA, United States); the *Actin* (100500082) gene was used as the internal control (Jian et al., 2008). The expression levels of nine *GmPP2A-B′′* genes were determined using the 2^–Δ^ ^Δ^ ^CT^ method. All primers are listed in Supplementary Table 2.

### Subcellular Localization Analysis

The full length coding sequence (CDS) of *GmPP2A-B′′71* was cloned into vector 16318h-GFP, containing a green fluorescent protein (GFP) reporter gene, and the recombinant vector was transformed into the Top10 strain of *Escherichia coli*. After sequencing, the GmPP2A-B′′71-GFP recombinant plasmid and mCherry vectors (the signals of mCherry proteins were located in the nucleus) harboring nuclear-localized proteins were co-transformed into *Arabidopsis* protoplasts by the PEG4000-mediated method (Yoo et al., 2007). GFP signals of protoplasts were observed using a laser confocal microscope (Carl Zeiss LSM 700, Germany) after 16 h of dark induction at room temperature.

### Transformation of *Arabidopsis* and Propagation of Positive Seedlings

The CDS of *GmPP2A-B′′71* was cloned into vector pCAMBIA1302 to construct recombinant vector pCAMBIA1302-*GmPP2A-B′′71*. After sequencing, the recombinant vector was transformed into *Agrobacterium tumefaciens* strain GV3101 by the freeze-thaw method. *Agrobacterium*-mediated inflorescence impregnation was used to generate transgenic *Arabidopsis* plants. *Arabidopsis* plants (ecotype Col-0) were grown under normal conditions (16:8 h light/dark photoperiod; 23°C; 50% relative humidity) until flowering. *A. tumefaciens* carrying the recombinant vector was cultivated in 50 mL of LB liquid culture medium with antibiotics (rifampicin and kanamycin). When the optical density (OD) of the culture reached 0.6–0.8, the culture was spun down and re-suspended in 1/2 MS liquid medium to generate the *Agrobacterium* impregnation solution to be used for *Arabidopsis* transformation, according to the methods described by Yu et al. (2018).

The T_0_ generation of transgenic *Arabidopsis* plants were screened on 1/2 MS solid medium supplemented with hygromycin (30 mg/L). After screening for 14 days, positive transgenic *Arabidopsis* seedlings were transferred into soil for growth. The T_3_ generation of transgenic *Arabidopsis* plants were used for qPCR analysis to determine the expression level of *GmPP2A-B′′71*. After identification, the T_4_ positive generation of transgenic *Arabidopsis* seedlings were used in experiments.

### Salt and Drought Tolerance of Transgenic *Arabidopsis* Plants

The seeds of three homozygous *GmPP2A-B′′71* transgenic *Arabidopsis* lines and wild-type (WT) plants were sown into flowerpots. Three weeks old of *GmPP2A-B′′71* transgenic *Arabidopsis* and WT plants were used for stress tolerance analysis. For salt tolerance experiments, the 3 week old plants were irrigated with a 250 mM NaCl solution. After 5 days of salt treatment, leaf samples were collected for measurement of relative electrolyte leakage. After 7 days of salt treatment, phenotypes were observed and survival rates were calculated. In addition, we used different concentrations of NaCl (100 and 125 mM) for salt stimulation to observe the root system of *GmPP2A-B′′71* transgenic and WT *Arabidopsis* seedlings. The seeds of *GmPP2A-B′′71* transgenic and WT *Arabidopsis* plants were disinfected by sodium hypochlorite (0.9%), transferred to 1/2 MS solid medium for 3 days of low temperature treatment (4–8°C), then transferred to normal growth conditions. After 7 days of growth, the seedlings of *GmPP2A-B′′71* transgenic and WT *Arabidopsis* plants were transferred onto 1/2 MS solid medium supplemented with different concentrations of NaCl (100 and 125 mM) for salt stress treatment. After 7 days of treatment, the fresh weight and root lengths were measured.

For drought experiments, watering was withheld from 3 week old *GmPP2A-B′′71* for 10 days, after which leaf samples were taken for measurement of relative electrolyte leakage. After 14 days of drought treatment, phenotypes were observed and the soil water content of each flowerpot was measured; the resulting data is presented in Supplementary Table 3. Survival rates were also calculated. The seeds of *GmPP2A-B′′71* transgenic and WT *Arabidopsis* plants were disinfected with sodium hypochlorite (0.9%), transferred to 1/2 MS solid medium for 3 days of low temperature treatment (4–8°C), then transferred to normal growth conditions. After 7 days of growth, the seedlings of *GmPP2A-B′′71* transgenic and WT *Arabidopsis* plants were transferred to 1/2 MS solid medium supplemented with different concentrations of PEG6000 (4 and 6% PEG6000) to simulate drought stress. After 7 days of treatment, the fresh weight and root lengths were measured. Experiments were repeated three times. Each experimental repetition included 5 groups, and each group contained 10 each of *GmPP2A-B′′71*-OE1, *GmPP2A-B′′71*-OE2, *GmPP2A-B′′71*-OE3, and WT (Col-0) plants. All plants of were cultured at 25°C under a 16:8 h light/dark photoperiod in a greenhouse.

### *Agrobacterium rhizogenes* Mediated Transformation of Hairy Root Soybean

Seeds of soybean variety “Williams 82” were sown into flowerpots. The CDS of *GmPP2A-B′′71* was cloned into the pCAMBIA3301 vector by homologous recombination to create the OE construct. To create the *GmPP2A-B′′71* RNA interference (RNAi) construct, 200 bp of the *GmPP2A-B′′71* CDS containing a hairpin structure was selected as the inducer sequence, artificially synthesized (BGI China), and cloned into the pCAMBIA3301 vector. The overexpressing-, interfering-, and empty-vector (EV-control) were transformed into *Agrobacterium rhizogenes* strain K599. The resulting *A. rhizogenes* strains were cultured for 3 days in a 28°C incubator, then used to transform soybean plants using the methods described by Kereszt et al. (2007). In brief, *A. rhizogenes* carrying the respective recombinant vectors were injected into 7 day old soybean plants, 1–2 mm below the cotyledonary node. After injection, soybean seedlings were kept in darkness for 12 h of induction, then grown under normal conditions (25°C; 16:8 h light/dark) for 3 weeks to encourage hairy root growth; after 3 weeks hairy roots grew up to about 5 cm. The part 1 cm below the inoculation site was removed, and soybean seedlings with hairy roots were transferred into new flowerpots to generate mature soybean plants with *GmPP2A-B′′71* transgenic hairy roots.

### Analysis of Salt and Drought Tolerance of Transgenic Soybean Plants

In salt tolerance experiments, EV-control and *GmPP2A-B′′71* transgenic soybean plants with hairy roots were irrigated with a 250 mM NaCl solution. After 3 days of salt treatment, 0.1 g of hairy root samples were collected. The malondialdehyde (MDA) and proline (Pro) contents of samples were measured using the MDA and Pro Assay kits (Solarbio, China), and the catalase (CAT) and peroxidase (POD) activities of the samples were measured using the Micro Catalase (CAT) and Micro Peroxidase assay kits (Solarbio, China), respectively, following the manufacturers protocols. After 1 week of salt stress induction, phenotypes were observed and chlorophyll content was measured.

In drought tolerance experiments, water was withheld from EV-control and *GmPP2A-B′′71* transgenic soybean plants with hairy roots for 10 days of drought treatment, after which hairy root samples were collected and the MDA and Pro contents, and CAT and POD activities were measured as described. After 14 days of drought treatment, phenotypes were observed and chlorophyll and soil water contents were measured. Soil water content data is presented in Supplementary Table 3. Experiments were repeated five times. Each experimental repetition included three groups, with each group containing five each of *GmPP2A-B′′71*-RNAi, EV-control, and *GmPP2A-B′′71*-OE soybean plants. All soybean plants were cultured at 25°C under a 16:8 h light/dark photoperiod in a greenhouse.

### Measurement of Soil Water Content

For drought tolerance experiments, soybean plants were grown in 15 cm diameter flowerpots containing 315.16 ± 0.1 g of soil (1:1 ratio of soil to vermiculite) and watered with 300 mL of water. To determine the soil water content of soil under normal growth conditions, soil samples were collected after 3 days of growth and the weight was recorded (W1), after which the samples were baked at 150°C until completely dry, and the weight was again recorded (W2); the soil water content was calculated using the following formula: (W1–W2) × 100/W1. After 14 days of no watering, soil samples were taken again to determine soil water content under drought conditions, as described for the samples taken under normal growth conditions.

### Nitro Blue Tetrazolium Staining

To assess the degree of ROS accumulation of *GmPP2A-B′′71*-RNAi, EV-control, and *GmPP2A-B′′71*-OE soybean plants under salt and drought treatments, root tips and leaves of stress-induced plants were collected and soaked in Nitro Blue Tetrazolium (NBT) solution for 30 min and overnight, respectively. After 30 min of staining, the stress-induced root tips were observed under the microscope (LEICA M165 FC, Germany). Leaves were transferred to a 7:3 (v/v) acetone/glycerol decolorization solution and boiled (100°C) for 1 h then observed under the integrated microscope.

### Measurement of Chlorophyll Content and Relative Electrolyte Leakage

To measure the chlorophyll content in transgenic soybean leaves, 0.1 g of leaf tissue was sampled from each transgenic soybean line (*GmPP2A-B′′71*-OE, EV-control, and *GmPP2A-B′′71*-RNAi), pulverized with a mortar and pestle, then incubated in 1 mL of 80% acetone solution for 12 h in darkness. The chlorophyll content of the samples was then measured using a Varioskan LUX enzyme-labeling measuring instrument (Thermo Fisher Scientific, United States) at wavelengths of 665 and 649 nm; the acetone solution alone was used as a blank control. Measurements were taken from three biological replicates.

A total of 0.1 g of hairy root samples were collected from the three soybean lines, cultivated under natural growth conditions and under drought and salt treatments, into a test tube containing 20 mL of distilled water, and then vacuumized for 30 min. The sample tubes were then stand for 2 h to measure the initial electric conductance (S1) (25°C). The hairy root samples were then heated at 100°C for 30 min, then allowed to cool to room temperature (25°C) to determine the final electric conductance (S2). The relative electrolyte leakage was evaluated as: REC = S1 × 100/S2.

### Statistical Analysis

SPSS 19.0 software (IBM Corp., Armonk, NY, United States) was used to compare the means of each treatment group using one-way ANOVA and *t*-tests. Differences were considered significant when at *p* < 0.05. Results are expressed as the mean ± standard deviation (SD).

## Results

### Identification of Soybean PP2A-B′′ Family Members

**FIGURE 1:**
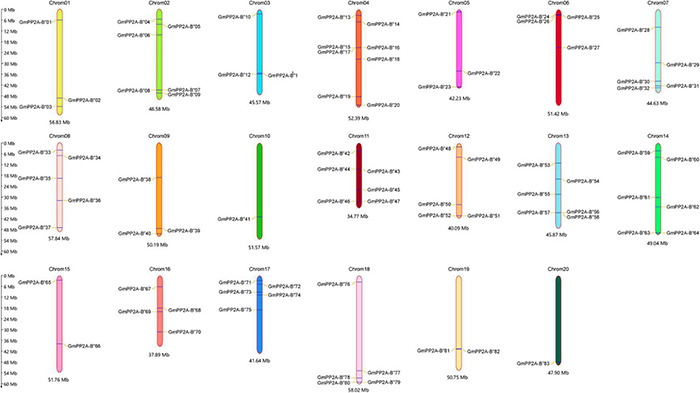
The location of 83 *GmPP2A-B′′* genes on the soybean chromosome. The scale bar on the left indicates the size of the chromosomes.

We identified 83 GmPP2A-B′′ members (Supplementary Table 1) and named *GmPP2A-B′′01* to *GmPP2A-B′′83* according to their chromosomal positions and Pfam databases (Figure 1). A total of 83 *GmPP2A-B′′* genes were distributed in 20 chromosomes of soybean, and chromosome 4 contained the largest number with 8 *GmPP2A-B′′* genes.

**FIGURE 2:**
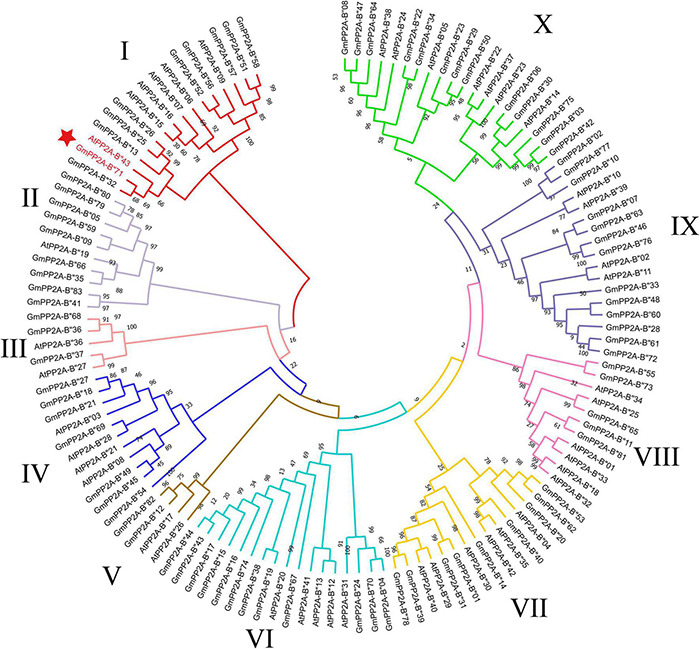
A total of 83 *GmPP2A-B′′s* and 43 *AtPP2A-B′′s* were used for multiple sequence alignment analysis to construct a phylogenetic tree with the method of ML. The 10 groups are represented by different colors.

Forty-three *AtPP2A-B′′* genes were identified in *Arabidopsis*, and *PP2A-B′′* family genes from soybean and *Arabidopsis* were used to construct a phylogenetic tree using the ML method with MEGA-X software. Our results showed that soybean PP2A-B′′ family members were divided into 10 subgroups (I–X) (Figure 2). Group X contained the largest number of *PP2A-B′′* member genes than other subgroups, including 13 *GmPP2A-B′′* genes and 7 *AtPP2A-B′′* genes. Group V had the least number of *PP2A-B′′* member genes; it contained two *GmPP2A-B′′* genes and two *AtPP2A-B′′* genes (Figure 2).

**FIGURE 3:**
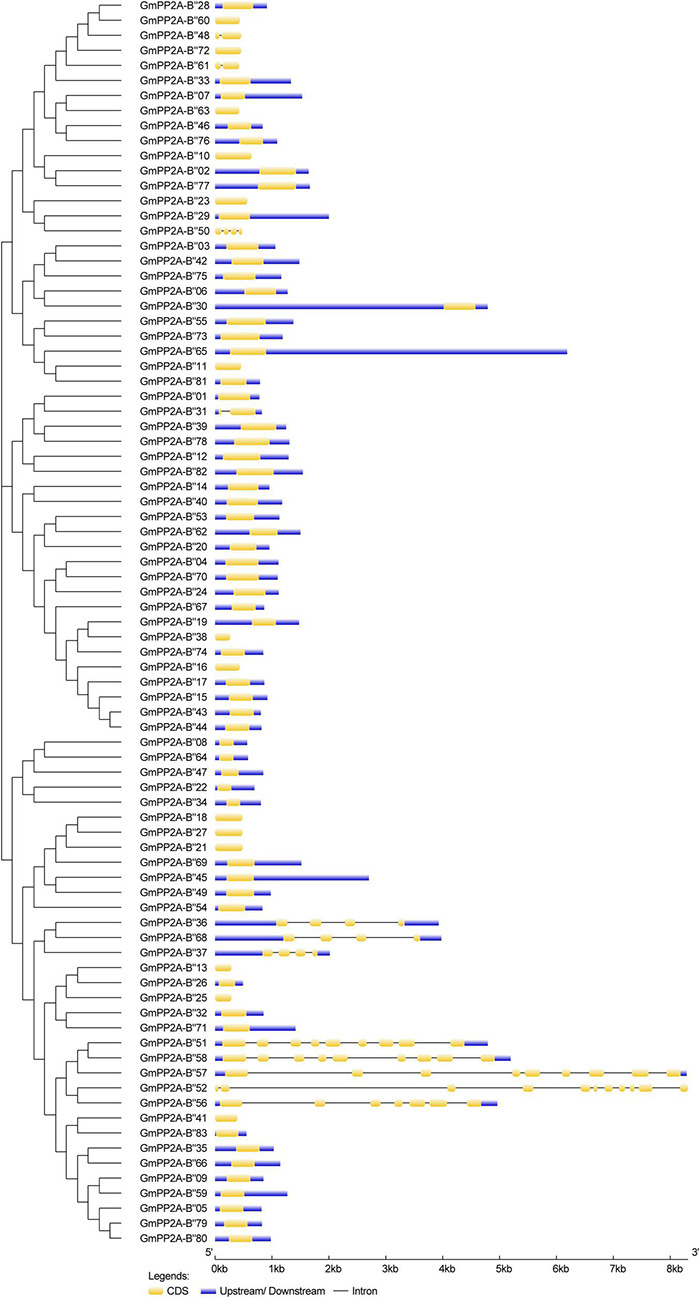
The gene structures of 83 *GmPP2A-B′′* family genes. The schematic diagram indicates the gene structure. Introns are indicated by black lines, exons are indicated by yellow boxes, and upstream/downstream are indicated by blue boxes. The lengths of introns and exons of each gene are displayed proportionally.

**FIGURE 4:**
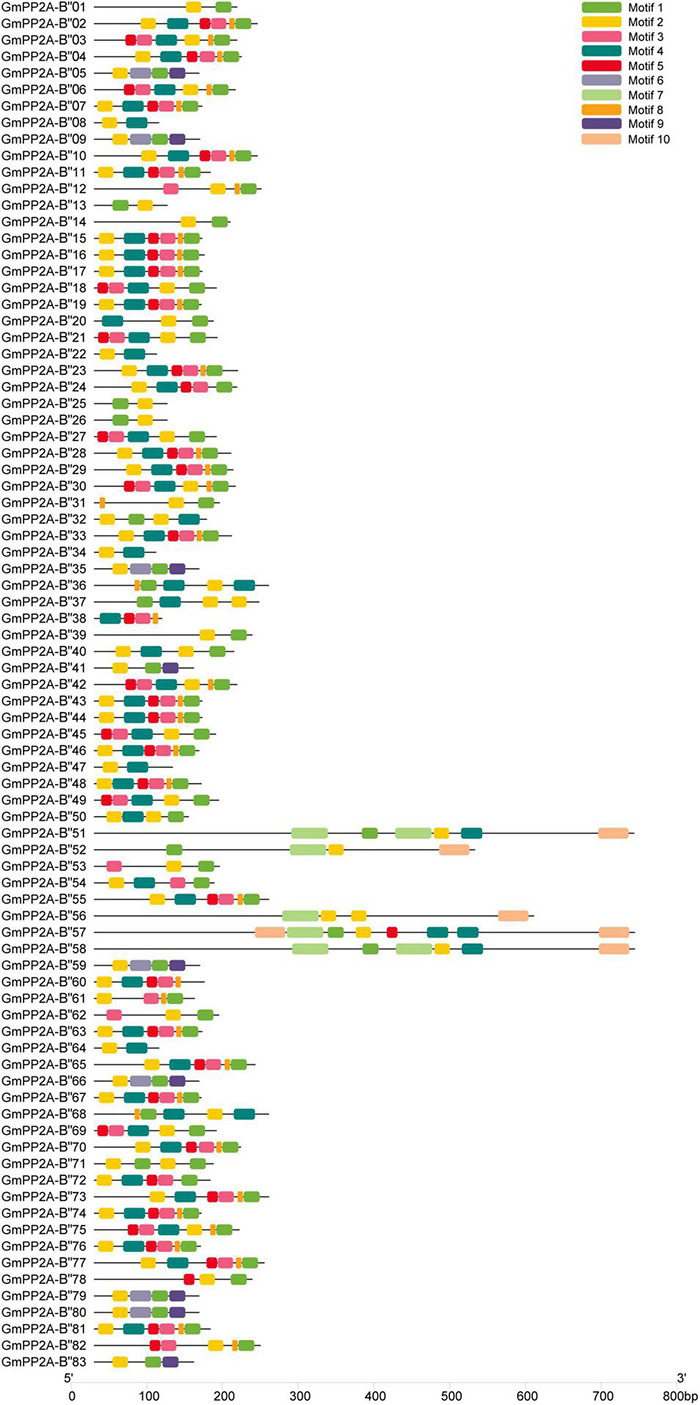
Putative motifs in each GmPP2A-B′′ protein. The phylogenetic tree was constructed using MEGA-X software. Conserved motifs were identified using MEME. Ten boxes of different colors represent different putative motifs. The scale at the bottom estimates the length of each protein.

The gene structures showed that 12 GmPP2A-B′′ members contained introns including *GmPP2A-B′′31/36/37/48/50/51/52/56/57/58/61/68* (Figure 3). We identified 10 motifs in 83 GmPP2A-B′′ family members, 11 GmPP2A-B′′ members contained 2 motifs (the least number of motifs), and GmPP2A-B′′57 contained the most motifs with 8 (Figure 4).

### Tissue-Specific Expression Patterns of *GmPP2A-B′′* Genes

**FIGURE 5:**
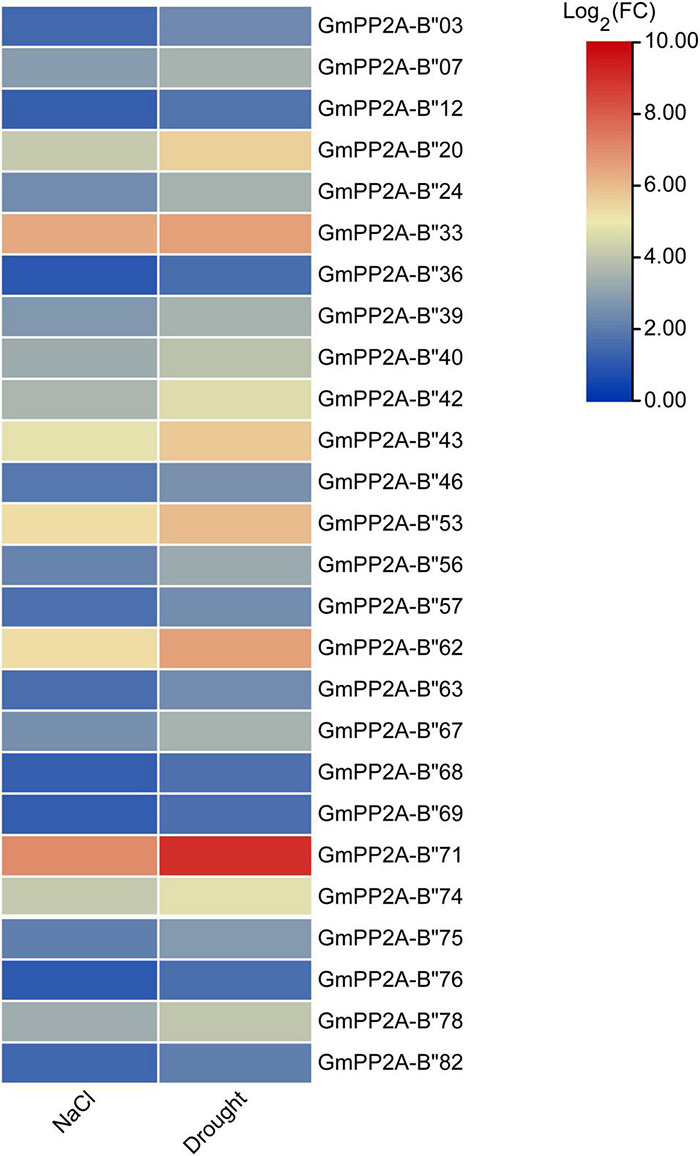
Heat map of the expression abundances (in log2-based transcriptome data) of 26 *GmPP2A-B′′* genes under drought and salt stress conditions. The expressions were represented by the different colors. Red means higher expression; blue means lower expression.

**FIGURE 6:**
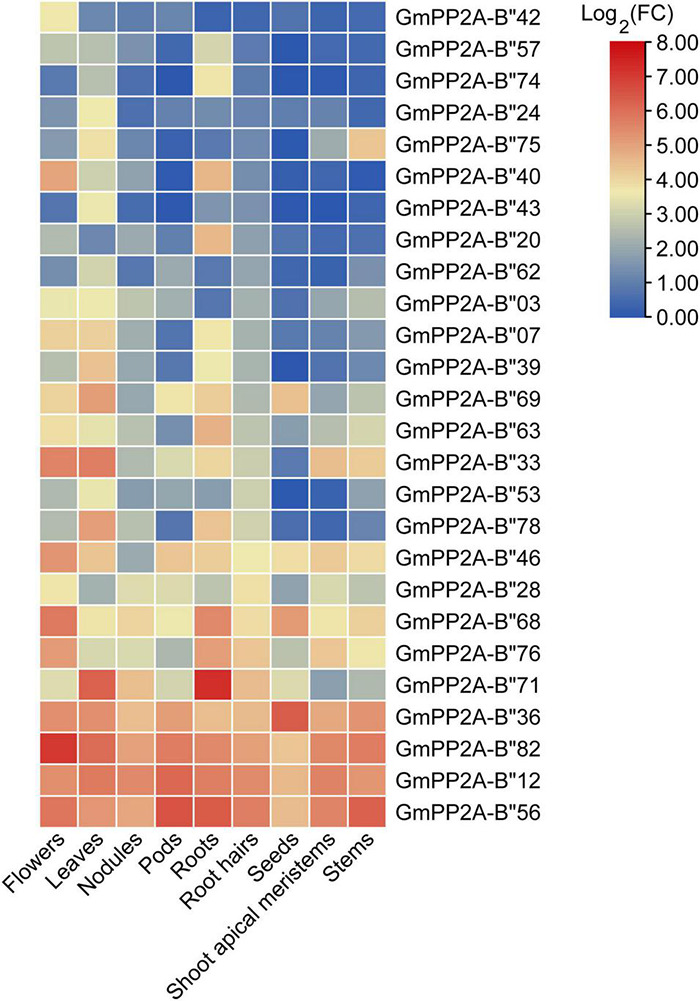
Heat map of the expression profiles of 26 *GmPP2A-B′′* genes in different soybean tissues. The expression data of 26 *GmPP2A-B′′* genes in different tissues were obtained from Phytozome. Expression levels in nine different tissues are shown: flowers, leaves, nodules, pods, roots, root hairs, seeds, shoot apical meristems, and stems.

To analyze the function of *GmPP2A-B′′* genes in plant stress tolerance, we performed RNA-seq analysis on soybean plants which were treated with drought, salt, or full irrigation conditions. RNA-seq analysis demonstrated 26 *GmPP2A-B′′* genes with significantly up-regulated expression under salt and drought stresses, respectively (Figure 5). These results indicated that 26 *GmPP2A-B′′* could be involved in response to abiotic stress. The expression levels of these 26 genes in different tissues were downloaded from the Phytozome website and the heat map was constructed by TBtools. Our results demonstrated that the expression levels of *GmPP2A-B′′71* in soybean roots and leaves were significantly higher than in other 25 *GmPP2A-B′′* genes. *GmPP2A-B′′82* had a higher expression level in flowers than the other 25 *GmPP2A-B′′* genes, and it also had high expression levels in the hairy roots, leaves, nodules, and shoots. Of these 26 *GmPP2A-B′′* genes, *GmPP2A-B′′36* had the highest expression in the seed, while *GmPP2A-B′′56* had higher expression levels in the pods, flowers, roots, hairy roots, and shoots than other tissues (Figure 6). *GmPP2A-B′′12* was highly expressed in the leaves, nodules, pods, and shoots. *GmPP2A-B′′33/46/68/69* were highly expressed in the leaves, pods, flowers, and leaves, respectively (Figure 6). As such, these *GmPP2A-B′′* genes were selected for further study.

### *Cis*-Acting Element Analysis of Nine *GmPP2A-B′′* Genes

**FIGURE 7:**
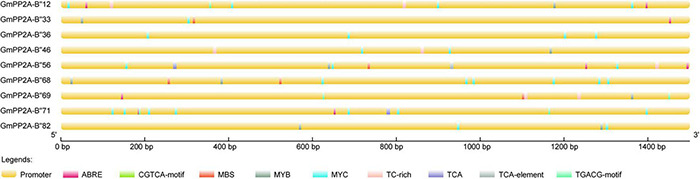
Distribution of *cis*-acting elements in the promoters of soybean *GmPP2A-B′′* genes. Promoters are indicated by yellow boxes and nine different-colored boxes represented nine different *cis*-acting elements. The scale at the bottom estimates the length of each protein.

To further analyze the relationships between these nine *GmPP2A-B′′* genes and abiotic stress responses, we analyzed 1500 bp upstream of the start codon in promoters of nine *GmPP2A-B′′* genes using PlantCARE. Our results indicated that some *cis*-acting elements, such as MYC, MYB, MBS, TC-rich, TCA, CGTCA-motif, and ABRE, are related to stress responses by analyzing their promoter sequence (Figure 7). Further analysis demonstrated that *GmPP2A-B′′12/33/46/56/68/71/82* all contained MYC *cis*-acting elements. Of the nine genes, *GmPP2A-B′′12/33/46/56/68/69/82* contained MYB-*cis*-acting elements, while *GmPP2A-B′′33/56/68/69* contained MBS *cis*-acting elements. *GmPP2A-B′′12*/*33/56*/*71*/*69* contained ABRE *cis*-acting elements. *GmPP2A-B′′36* and *GmPP2A-B′′71* had TCA *cis*-acting elements, and *GmPP2A-B′′12*, *GmPP2A-B′′69*, and *GmPP2A-B′′71* had CGTCA-motif elements (Figure 7). These results further indicate that these nine genes are related to abiotic stress responses.

### Expression Pattern Analysis of Nine *GmPP2A-B′′* Genes

**FIGURE 8:**
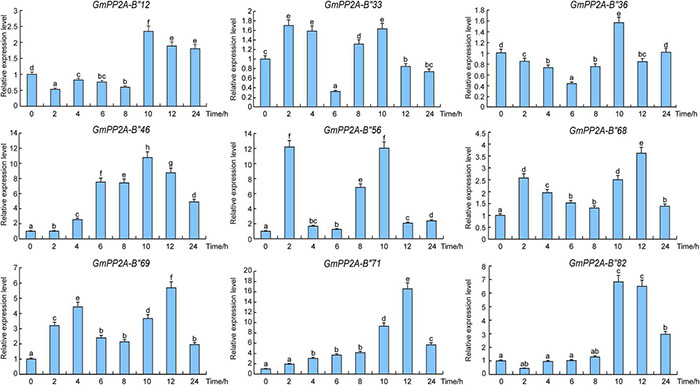
Expression patterns of *GmPP2A-B′′* genes under salt stress conditions. Expression levels of nine *GmPP2A-B′′* genes were measured using qPCR at different times of NaCl treatment and the expression levels were determined using the 2^–Δ^ ^Δ^ ^CT^ method. qPCR data were normalized using control *Actin* (100500082) as the reference gene and were displayed relative to 0 h. The *x*-axes show the duration of treatment and the *y*-axes depict relative expression levels (error bars indicate SD). The data are shown as the means of three biological replicates ± SD. Different letters indicate significant differences at *p* < 0.05 according to two-way ANOVA (Duncan’s multiple range test). Different letters indicate significant differences at *p* < 0.05 according to two-way ANOVA (Duncan’s multiple range test).

**FIGURE 9:**
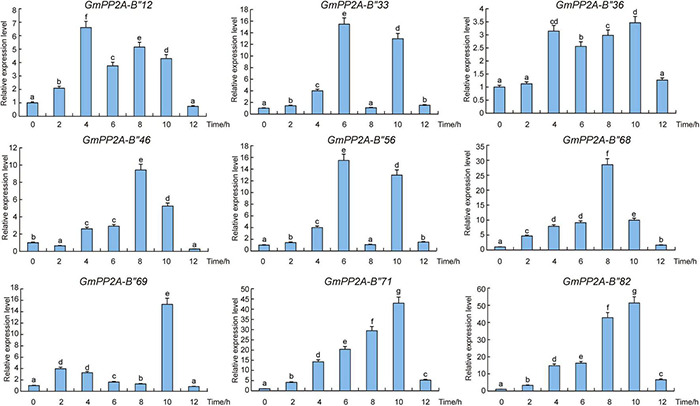
Expression patterns of *GmPP2A-B′′* genes under drought stress. Expression levels of nine *GmPP2A-B′′* genes were measured using qPCR at different times of drought treatment and the expression levels were determined using the 2^–Δ^ ^Δ^ ^CT^ method. qPCR data were normalized using control *Actin* (100500082) as the reference gene and were displayed relative to 0 h. The *x*-axes show the duration of treatment and the *y*-axes depict relative expression level (error bars indicate SD). The data are shown as the means of three biological replicates ± SD. Different letters indicate significant differences at *p* < 0.05 according to two-way ANOVA (Duncan’s multiple range test).

In order to confirm whether the function of *GmPP2A-B′′* was related to stress resistance of soybean, the expression patterns of these *GmPP2A-B′′* genes were analyzed *via* qPCR. Our results demonstrated that these *GmPP2A-B′′* genes were differentially induced under drought and salt stress conditions. For example, drought can induce these *GmPP2A-B′′* genes to up-regulate expression. Under drought stress, *GmPP2A-B′′12*, *GmPP2A-B′′33*, *GmPP2A-B′′36*, and *GmPP2A-B′′68* were induced, while the expression levels of *GmPP2A-B′′56* reached a peak after 2 h of drought treatment. *GmPP2A-B′′46* and *GmPP2A-B′′82* could be significantly induced after 10 h of drought treatment. Moreover, the expression levels of *GmPP2A-B′′69* and *GmPP2A-B′′71* reached peaks at 12 h of drought treatment (Figure 8). Similarly, salt can significantly induce nine *GmPP2A-B′′* genes to up-regulate their expression. *GmPP2A-B′′12* and *GmPP2A-B′′36* can be significantly induced by salt at 4 h and reached their peaks at the same time (Figure 9). The expression levels of *GmPP2A-B′′33* and *GmPP2A-B′′56* peaked after 6 h of salt treatment, and the expression levels of *GmPP2A-B′′46* and *GmPP2A-B′′68* significantly increased after 8 h of salt treatment (Figure 9). In addition, the expression levels of *GmPP2A-B′′69*, *GmPP2A-B′′71*, and *GmPP2A-B′′82* were significantly induced under salt stress conditions and peaked after 10 h of salt treatment (Figure 9). Of these nine genes, *GmPP2A-B′′71* was significantly induced under both drought and salt treatments, leading us to select *GmPP2A-B′′71* genes for further study.

### GmPP2A-B′′71 Protein Was Located in the Nucleus and Cytoplasm

**FIGURE 10:**
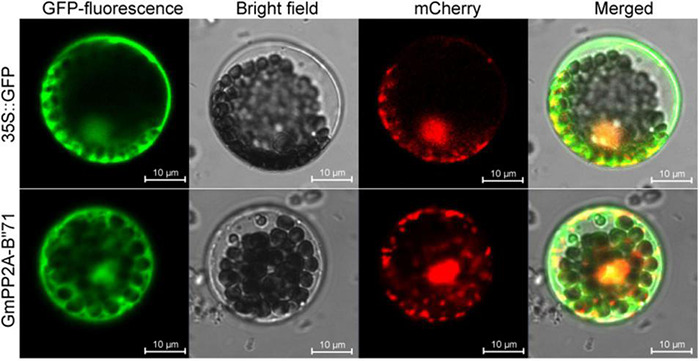
Subcellular localization of GmPP2A-B′′71-GFP fusion protein. 35S:GFP was used as a control. The scale bar of 35S:GFP and GmPP2A-B′′71-GFP indicate 10 μm.

To analyze the molecular characteristics of the GmPP2A-B′′71 protein, we cloned the coding sequence length (CDS) sequence of *GmPP2A-B′′71* and fused it into the GFP to form a GmPP2A-B′′71-GFP recombinant vector. The recombinant vector was then transferred into *Arabidopsis* leaf protoplast cells using the PEG4000-mediated method. Our results demonstrated that GmPP2A-B′′71-GFP was located in the nucleus and cytoplasm (Figure 10), which similar to GFP.

### *GmPP2A-B′′71* Can Improve Drought and Salt Tolerance in Transgenic *Arabidopsis*

**FIGURE 11:**
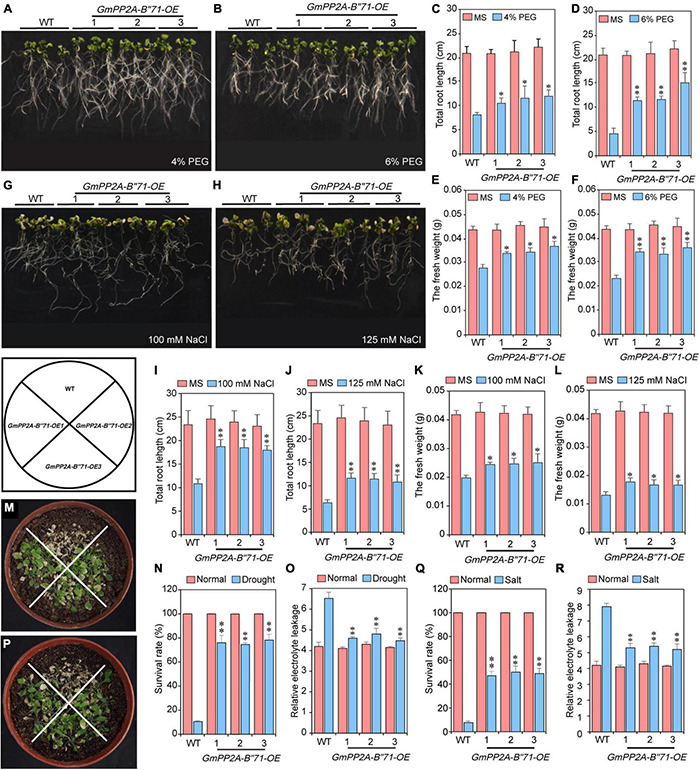
Analysis of *GmPP2A-B′′71* functions in *Arabidopsis* under normal and different concentrations of PEG and NaCl stress conditions. **(A,B)** Phenotypic analysis of the *GmPP2A-B′′71* transgenic *Arabidopsis* lines and WT plants under different concentrations of PEG treatment. **(C,D)** Total root length analysis of the *GmPP2A-B′′71* transgenic *Arabidopsis* lines and WT plants under normal and different concentrations of PEG treatment. **(E,F)** The fresh weight of *GmPP2A-B′′71* transgenic *Arabidopsis* lines and WT plants under different concentrations of PEG conditions. **(G,H)** Phenotypic analysis of the *GmPP2A-B′′71* transgenic *Arabidopsis* lines and WT plants under different concentrations of NaCl treatment. **(I,J)** Total root length analysis of the *GmPP2A-B′′71* transgenic *Arabidopsis* lines and WT plants under normal and different concentrations of NaCl treatment. **(K,L)** The fresh weight of *GmPP2A-B′′71* transgenic *Arabidopsis* lines and WT plants under normal and different concentrations of NaCl conditions. **(M)** Phenotypes of transgenic *Arabidopsis* lines and WT plants under drought conditions. **(N)** The survival rates of transgenic *Arabidopsis* lines and WT plants under normal and drought stress conditions. **(O)** Relative electrolyte leakage of transgenic *Arabidopsis* and WT under drought normal and stress conditions. **(P)** Phenotypes of transgenic *Arabidopsis* lines and WT plants under salt conditions. **(Q)** The survival rates of transgenic *Arabidopsis* lines and WT plants under normal and salt stress conditions. **(R)** Relative electrolyte leakage of transgenic *Arabidopsis* and WT under normal and salt stress conditions. *p < 0.05 and ^**^p < 0.01.

To investigate whether *GmPP2A-B′′71* can improve the tolerance of transgenic plants, *GmPP2A-B′′71* was inserted into the *Arabidopsis* genome *via A. rhizogenes*-mediated transformation, which resulted in three independent transgenic lines, including *GmPP2A-B′′71-OE1*, -*OE2*, and -*OE3*. Transgenic *Arabidopsis* seeds of T_4_ generation were selected for subsequent experiments. We found that under 4 and 6% PEG the total root length and fresh weight of transgenic *Arabidopsis* lines were significantly higher than WT (Figures 11A–F). Under normal conditions, the total root length and fresh weight of three transgenic *Arabidopsis* lines did not differ from WT plants (Supplementary Figure 4). Under drought conditions, we found that the survival rate and relative electrolyte leakage of the three transgenic *Arabidopsis* lines were significantly higher than WT (Figures 11M–O), while there was no significant difference between transgenic *Arabidopsis* lines and WT plants under normal conditions (Supplementary Figure 5). These results indicate that transgenic *Arabidopsis* have higher drought tolerance than WT plants (Figure 11).

We also used NaCl to treat transgenic *Arabidopsis* lines and WT plants. Similarly, total root length and the fresh weight of WT and transgenic *Arabidopsis* under salt stress were measured. Our results demonstrated that under 100 mM and 125 mM NaCl conditions, the total root length of transgenic *Arabidopsis* was significantly longer than WT, and the fresh weight of transgenic *Arabidopsis* was also significantly higher than WT, while under normal conditions, there was no significant difference between WT and transgenic *Arabidopsis* lines in root length and fresh weight (Figures 11G–L). We also calculated the survival rate and relative electrolyte leakage of WT and transgenic *Arabidopsis* lines under salt stress. We found that under 100 and 125 mM NaCl conditions, the survival rate and relative electrolyte leakage of transgenic *Arabidopsis* lines were significantly higher than WT (Figures 11P–R). These results indicate that transgenic *Arabidopsis* lines had higher salt tolerance than WT plants (Figure 11).

### Overexpression of *GmPP2A-B′′71* in Soybean Hairy Roots Can Improve Drought Tolerance in Soybean Plants

To further analyze the relationship between *GmPP2A-B′′71* and plant drought tolerance, we used the *A. rhizogenes*-mediated transformation method to generate the transgenic *GmPP2A-B′′71* soybean hairy root composite plants. We obtained three different types of transgenic soybean hairy root composite plants, and named them *GmPP2A-B′′71*-RNAi, EV-control, and *GmPP2A-B′′71*-OE composite plants. We used composite plant materials from these three different kinds of transgenic soybean hairy roots to analyze how the *GmPP2A-B′′71* genes respond to plant drought tolerance. For drought tolerance analysis, the *GmPP2A-B′′71*-RNAi, EV-control, and *GmPP2A-B′′71*-OE soybean plants that were cultured under normal conditions were transferred to drought conditions for 2 weeks. After 14 days of drought treatment, the leaves of most *GmPP2A-B′′71*-RNAi plants turned yellow and severely wilted, which indicates severe dehydration, while the EV-control lines displayed moderate wilting. However, the growth activity of *GmPP2A-B′′71*-RNAi plants declined, while *GmPP2A-B′′71*-OE plants were still upright, and the leaves of *GmPP2A-B′′71*-OE plants were green and displayed stronger growth activity compared to the *GmPP2A-B′′71*-RNAi and EV-control plants. This indicates that overexpression of *GmPP2A-B′′71* could improve plant tolerance to drought stress.

**FIGURE 12:**
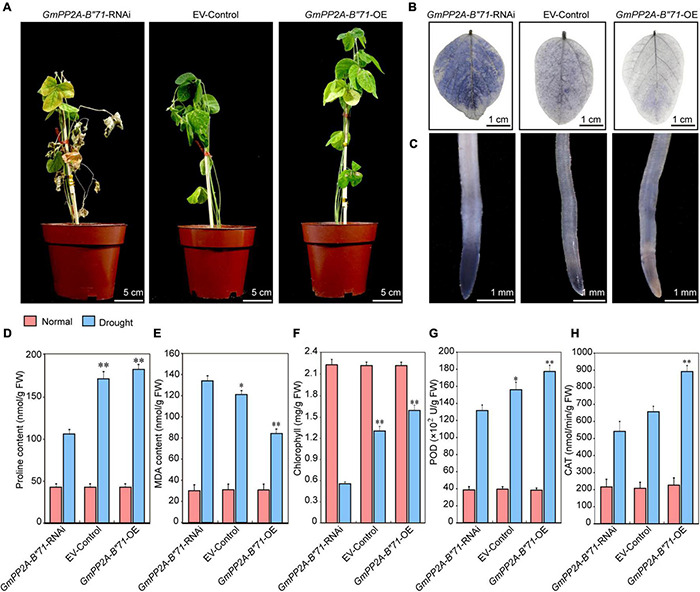
Analysis of the function of soybean *GmPP2A-B′′71* under drought stresses. **(A)** Phenotype analysis of *GmPP2A-B′′71*-RNAi, EV-Control, and *GmPP2A-B′′71*-OE plants under drought stress conditions. The scale bar indicates 5 cm. **(B)** NBT staining of *GmPP2A-B′′71*-RNAi, EV-Control, and *GmPP2A-B′′71*-OE plant leaves under drought stress conditions. The scale bar indicates 1 cm. **(C)** NBT staining of *GmPP2A-B′′71*-RNAi, EV-Control, and *GmPP2A-B′′71*-OE plant root tips under drought stress conditions. The scale bar indicates 1 mm. **(D–H)** The proline **(D)**, MDA **(E)**, chlorophyll **(F)**, POD **(G)**, and CAT **(H)** contents of *GmPP2A-B′′71*-RNAi, EV-Control, and *GmPP2A-B′′71*-OE plants under drought stress conditions. *p < 0.05 and ^**^p < 0.01.

Abiotic stress can induce a high level of ROS (Mhamdi and Van Breusegem, 2018). To verify the relationship between plant tolerance, ROS, and *GmPP2A-B′′71*, we performed Nitro Blue Tetrazolium (NBT) staining analysis to observe the ROS levels of the *GmPP2A-B′′71*-RNAi, EV-control, and *GmPP2A-B′′71*-OE soybean plants under drought treatment. Our results demonstrated that the leaves and root tips of *GmPP2A-B′′71*-OE plants had lower ROS levels with a lighter color of NBT staining than EV-control plants, indicating that *GmPP2A-B′′71* overexpressed soybeans had stronger ROS-scavenging ability than EV-control plants under drought conditions. *GmPP2A-B′′71*-RNAi plants presented the opposite phenotype, with higher ROS levels than EV-control plants. We further measured the chlorophyll content in the leaves and the Pro, MDA, POD, and CAT contents in the roots of *GmPP2A-B′′71*-RNAi, EV-control, and *GmPP2A-B′′71*-OE soybean plants under drought conditions. Our results demonstrated that Pro, chlorophyll, POD, and CAT contents in *GmPP2A-B′′71*-OE soybean plants were higher than in EV-control plants, while the MDA content in *GmPP2A-B′′71*-OE soybean plants was lower than in EV-control plants. However, *GmPP2A-B′′71*-RNAi plants had lower Pro, chlorophyll, POD, and CAT contents and higher MDA contents than EV-control plants (Figure 12). These results further confirmed our hypothesis that overexpression of *GmPP2A-B′′71* in soybean hairy roots can enhance plant tolerance to drought stress conditions.

### Overexpression of *GmPP2A-B′′71* in Soybean Hairy Roots Improve Salt Tolerance in Soybean

**FIGURE 13:**
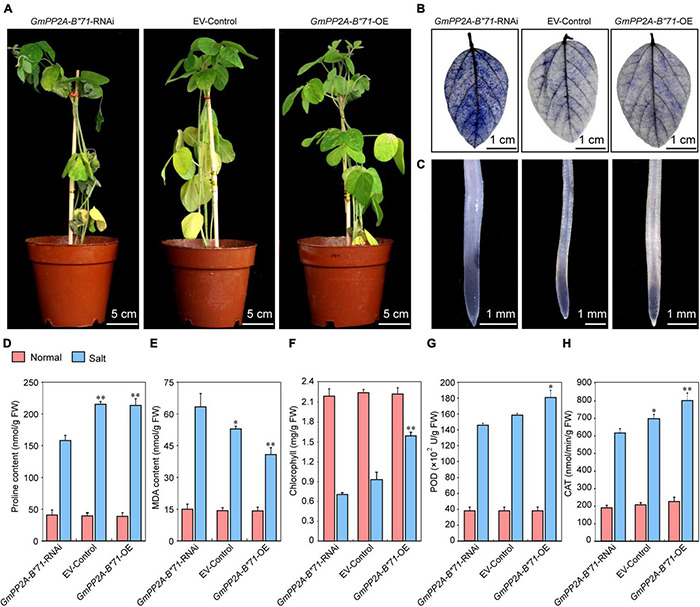
Analysis of the function of soybean *GmPP2A-B′′71* under salt stress conditions. **(A)** Phenotype analysis of *GmPP2A-B′′71*-RNAi, EV-Control, and *GmPP2A-B′′71*-OE plants under salt stress conditions. The scale bar indicates 5 cm. **(B)** NBT staining of the leaves of *GmPP2A-B′′71*-RNAi, EV-Control, and *GmPP2A-B′′71*-OE plants under salt treatment. The scale bar indicates 1 cm. **(C)** NBT staining of the root tips of *GmPP2A-B′′71*-RNAi, EV-Control, and *GmPP2A-B′′71*-OE plants under salt stress conditions. The scale bar indicates 1 mm. **(D–H)** The proline **(D)**, MDA **(E)**, chlorophyll **(F)**, POD **(G)**, and CAT **(H)** content of *GmPP2A-B′′71*-RNAi, EV-Control, and *GmPP2A-B′′71*-OE plants under salt stress conditions. *p < 0.05 and ^**^p < 0.01.

To further verify the relationships among plant stress tolerance, ROS, and *GmPP2A-B′′71*, we applied salt treatment for the *GmPP2A-B′′71*-RNAi, EV-control, and *GmPP2A-B′′71*-OE soybean plants by administering 250 mM NaCl. After 1 week of treatment, the leaves of the *GmPP2A-B′′71*-OE plants were still green and more unfurled than the EV-control plants, while the leaves of *GmPP2A-B′′71*-RNAi plants turned yellow and were more wilted than the EV-control plants. NBT staining results demonstrated that the degree of leaf staining area of *GmPP2A-B′′71*-OE plants was smaller and lighter than the EV-control plants, however, the *GmPP2A-B′′71*-RNAi plants had the opposite phenotype with severe wilting compared with EV-control plants. Similarly, the root tip staining results demonstrated that *GmPP2A-B′′71*-OE plants had lower ROS levels and a lighter color of NBT staining than EV-control plants, however, *GmPP2A-B′′71*-RNAi plants had the stronger colors of NBT staining with higher ROS levels than EV-control plants. This indicates that the overexpression of *GmPP2A-B′′71* in soybean plants can improve its salt tolerance. To further verify our conclusion, we measured the chlorophyll content of *GmPP2A-B′′71*-RNAi, EV-control, and *GmPP2A-B′′71*-OE soybean plant leaves and the contents of Pro, MDA, POD, and CAT in soybean hairy roots under salt treatment. Our results demonstrated that the contents of Pro, chlorophyll, POD, and CAT in *GmPP2A-B′′71*-OE soybean plants were higher than in EV-control and *GmPP2A-B′′71*-RNAi plants and that the MDA content in *GmPP2A-B′′71*-OE soybean plants was lower than in *GmPP2A-B′′71*-RNAi and EV-control plants (Figure 13). These results indicate that *GmPP2A-B′′71* contributes to salt stress tolerance in soybean plants.

### Overexpression of *GmPP2A-B′′71* Enhances Expression Levels of Stress-Responsive Genes

**FIGURE 14:**
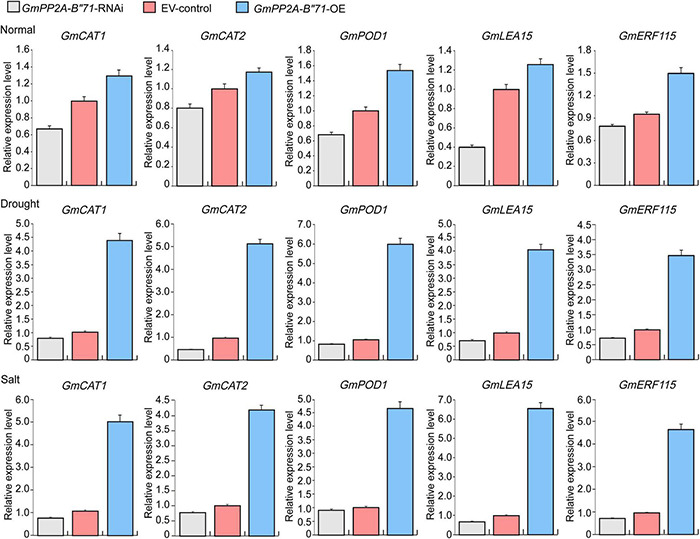
The expression levels of ROS scavenging- and stress-related genes in *GmPP2A-B′′71*-RNAi, EV-control, and *GmPP2A-B′′71*-OE soybean plants. Gray bar indicates *GmPP2A-B′′71*-RNAi; red bar indicates EV-control; blue bar indicates *GmPP2A-B′′71*-OE.

According to our NBT staining analysis, we found that *GmPP2A-B′′71*-OE plants had lower ROS levels than *GmPP2A-B′′71*-RNAi and EV-control plants and thus speculated that *GmPP2A-B′′71*-OE plants likely improve plant stress tolerance through the scavenging of ROS. To verify our hypothesis, we measured the expression levels of scavenging of ROS-related genes in *GmPP2A-B′′71*-RNAi, EV-control, and *GmPP2A-B′′71*-OE soybean plants using qPCR. Our results demonstrated that *GmCAT1*, *GmCAT2*, and *GmPOD* genes were significantly up-regulated in *GmPP2A-B′′71*-OE soybean plants and significantly down-regulated in *GmPP2A-B′′71*-RNAi plants. This indicates that *GmPP2A-B′′71* could regulate the ROS scavenging pathway. Additionally, some genes related to stress response, such as *GmLEA15* and *GmERF115*, were also significantly up-regulated in *GmPP2A-B′′71*-OE soybean plants (Figure 14). These results indicate that overexpression of *GmPP2A-B′′71* can increase the expression of stress response genes to improve plant stress tolerance.

## Discussion

Major plant serine/threonine protein phosphatases belong to the phosphoprotein phosphatase (PPP) family (Uhrig et al., 2013). PP2A, a rich iso-trimeric serine/threonine phosphatase (Tompa et al., 2014; Kataya et al., 2015; Qureshi et al., 2015), likes other PPPs, and the conserved domain short linear motifs (SLiMs) are located in its intrinsically disordered region (IDR) (Tompa et al., 2014; Wang et al., 2016). In our study, 83 *PP2A-B′′* genes were distributed on 20 chromosomes in soybean and divided into 10 subfamilies (Figure 2). Gene structure analysis demonstrated that *GmPP2A-B′′48* and *GmPP2A-B′′61* had similar intron structures and were divided into Group IX, while similar gene structure characteristics were discovered among *GmPP2A-B′′36*, *GmPP2A-B′′68*, *GmPP2A-B′′51*, and *GmPP2A-B′′58* (Figures 2, 3). Motif analysis showed GmPP2A-B′′ family members contained the same Motifs in the amino acid structure were divided into the same groups. For example, GmPP2A-B′′15, GmPP2A-B′′16, and GmPP2A-B′′17 had a similar Motif structure and were classified as Group VI (Figure 4). These results indicate that gene structure and protein modification might be important indicators for phylogenetic analysis. Further analysis demonstrated 26 *GmPP2A-B′′* genes were significantly induced in our drought- and salt-induced soybean transcriptome data (Figure 5). Of these 26 *GmPP2A-B′′* genes, we screened 9 *GmPP2A-B′′* genes for qPCR analysis based on tissue-specific expression analysis. Among these nine *GmPP2A-B′′* genes, *GmPP2A-B′′12*, *GmPP2A-B′′33*, *GmPP2A-B′′56*, *GmPP2A-B′′69*, and *GmPP2A-B′′71* had ABRE-*cis*-acting elements that reportedly play an important role in the ABA pathway (Yang et al., 2020) and have high expression levels under drought and salt stresses (Figures 7–9). These results indicate that these five *GmPP2A-B′′* genes could be associated with the ABA pathway and stress response. Additionally, we found that *GmPP2A-B′′36* and *GmPP2A-B′′68* had high homology and classified as Group III, they can all be induced by drought and salt stress. Similarly, both *GmPP2A-B′′56* and *GmPP2A-B′′71* were significantly induced under stress conditions and classified into Group I with the high homology (Figures 2, 8, 9), which suggested that they had conservation in the function to plant abiotic stress response.

As the climate changes, salt and drought stress will be the primary factors affecting soybean yield (Cui et al., 2019; Gao et al., 2020). Plants can respond to environmental stress through a series of signal regulation networks (Rasool et al., 2014), and the plant mevalonate (MVA) pathway plays an important role in plant stress tolerance. The *HMG-CoA reductase (HMGR)* gene is a key regulator in the MVA pathway, which is regulated by several endogenous signals and external stimuli (Chappell, 1995). Under normal conditions, PP2A-B′′β inhibits *HMGR* transcription, though PP2A-B′′α can regulate the transcription, translation, and activity levels of HMGR during plant response to salt stress (Leivar et al., 2011). These results indicate that the PP2A family participates in plant response to abiotic stress response. A novel Ca^2+^-binding protein, named AtCP1 (AtPP2A-B′′43) has a high degree of amino acid sequence homology to the Ca^2+^-binding loops of the EF-hands of calmodulin, and the expression levels of the *AtPP2A-B′′* genes can be induced by NaCl treatment (Jang et al., 1998). Our results demonstrated that *GmPP2A-B′′71* has high homology with *AtPP2A-B′′43* (Supplementary Figure 2) and can be induced by salt stress. These results further indicate that *GmPP2A-B′′71* participated in plant response to salt stress. In addition, some studies have demonstrated the key role of redox homeostasis in plant development, ROS production, and ROS-related signals on the growth of various plant tissues (Mhamdi and Van Breusegem, 2018). Abiotic stress can induce high ROS levels (Mhamdi et al., 2010). Recent research demonstrated that PP2A proteins are involved in salt tolerance and plant oxidative stress (Mathe et al., 2019, 2021). However, the relationship between PP2A proteins, ROS, and plant stress response was still unclear. Our study revealed that *GmPP2A-B′′71*-OE plants had lower ROS levels than EV-control plants under drought and salt stresses and that *GmPP2A-B′′71*-RNAi plants had higher ROS levels compared with EV-control plants (Figure 11). This indicates that *GmPP2A-B′′71* could be involved in the ROS regulation pathway and help to improve plant stress tolerance.

Reactive oxygen species pathways in plants can be divided into ROS synthesis pathways and ROS scavenging pathways. However, the most important way to improve plant stress tolerance is to improve ROS scavenging capacity (Baxter et al., 2014). Certain key genes play important roles in ROS scavenging capacity and plant stress tolerance. For example, CAT is an important enzyme involved in H_2_O_2_-metabolizing in plants (Mhamdi et al., 2010). The deficient function of CAT contributes to increased levels of peroxide (H_2_O_2_), which gives rise to an imbalance in ROS homeostasis. Overexpression of CAT removes the excess H_2_O_2_, which can maintain a steady pattern of ROS to mediate plant growth regulation (Vandenabeele et al., 2004). Harsh environmental conditions such as salt, drought, and heat can induce high ROS levels, while CAT can be induced in plants to improve stress tolerance by removing ROS (Mhamdi et al., 2010). POD is another important plant enzyme and can catalyze H_2_O_2_ to reduce its oxidative activity, maintaining plant growth homeostasis. The study demonstrates that stress-induced high POD expression levels contribute to the protection of a plant’s callus cells (Zhang et al., 2019). These results revealed that CAT and POD plants are critical to maintaining ROS homeostasis under stress conditions. The late embryogenesis abundant (LEA) protein is an important protein family and was highly enriched during late seed development. Research showed that the LEA protein is related to plant drought tolerance, and can be induced by high temperatures, salt, and cold temperatures (Poonia et al., 2020). When plants were exposed to abiotic stress conditions, resulting in the decreased cellular water content (Morales et al., 2003), LEA proteins were induced to protect the cell water content and prevent the intercellular dehydration caused by protein denaturation (Kovacs et al., 2008). Additionally, some study demonstrated that overexpression of LEAs in different plants can enhance transgenic plant stress tolerance. The overexpression of *NtLEA7-3* in *Arabidopsis* promotes drought and salt stress tolerance of transgenic *Arabidopsis* (Gai et al., 2011). A recent study showed that LEA proteins were associated with antioxidant activity and involved in the ROS pathway in plants (Poonia et al., 2020). These results indicate the importance of LEA in plant tolerance to abiotic stress. Plant transcription factors are regulators that play important roles in plant response to abiotic stresses. Of these plant transcription factors, the AP2/ERF transcription factor family regulates plant developmental processes and various kinds of environmental stress responses (Xu et al., 2011). A recent study found that the AP2/ERF transcription factor gene *AtERF115* can mediate the ROS signaling and maintain the root stem and root growth through phytosulfokine (PSK) peptide incorporation (Kong et al., 2018). This indicates the relationship between AP2/ERF transcription factors and the ROS signaling. The research revealed that LEA, CAT, POD, and ERF family gene were crucial in inhibiting accumulation of ROS under stress conditions. In addition, our study showed that under salt and drought stresses, *GmPP2A-B′′71*-OE plants presented higher stress tolerance and lower ROS levels than EV-control plants, and the qPCR results demonstrated that the expression levels of *GmLEA15*, *GmCAT1*, *GmPOD*, and *GmERF115* genes in *GmPP2A-B′′71*-OE plants were significantly enhanced compared with those in EV-control plants, while the expression levels of these genes decreased in *GmPP2A-B′′71*-RNAi plants. These results imply that *GmPP2A-B′′71* gene maybe affect the expression of transcriptional regulation factor which was related with ROS signaling, such as *GmERF115*, to mediate regulation of critical gene transcription that was involved in ROS scavenging. Together, these results indicate that *GmPP2A-B′′71* can enhance plant stress tolerance by regulating the ROS scavenging pathway, making it a candidate gene for plant tolerance to stress.

## Data Availability Statement

The original contributions presented in the study are included in the article/Supplementary Material, further inquiries can be directed to the corresponding authors.

## Author Contributions

Z-SX coordinated the project, conceived and designed the experiments, and edited the manuscript. YX performed the experiments and wrote the first draft. X-HF, QW, Z-GY, and X-WS generated and analyzed the data. Y-ZM and JM reviewed and contributed valuable discussions. Y-ZM coordinated the project. All authors have read and approved the final manuscript.

## Conflict of Interest

The authors declare that the research was conducted in the absence of any commercial or financial relationships that could be construed as a potential conflict of interest.

## Publisher’s Note

All claims expressed in this article are solely those of the authors and do not necessarily represent those of their affiliated organizations, or those of the publisher, the editors and the reviewers. Any product that may be evaluated in this article, or claim that may be made by its manufacturer, is not guaranteed or endorsed by the publisher.

## Footnotes

1
https://phytozome.jgi.doe.gov/pz/portal.html

2
https://web.expasy.org/protparam/

3
http://mg2c.iask.in/mg2c_v2.0/

4
http://gsds.cbi.pku.edu.cn/

5
http://meme-suite.org/tools/meme

6
https://www.soybase.org/
