# Supplementary material for: Genomic Analysis of Soybean PP2A-B′′ Family and Its Effects on Drought and Salt Tolerance
Source: Front Plant Sci. 2022 Feb 2;12:784038. doi: 10.3389/fpls.2021.784038 (PMC8847135; doi:10.3389/fpls.2021.784038)
Supplement: Supplementary file 1 [file Data_Sheet_1.pdf]

Genomic analysis of soybean PP2A-B" family and its effects on drought and salt tolerance

Running title: *GmPP2A-B"71* Confers Plant Stress Tolerance

Yang Xiong[1,2†], Xu-Hong Fan[3†], Qiang Wang[4], Zheng-Gong Yin[4], Xue-Wen Sheng[5], Jun Chen[2], Yong-Bin Zhou[2], Ming Chen[2], You-Zhi Ma[2], Jian Ma[1*], Zhao-Shi Xu[1,2*]

1 College of Agronomy, Jilin Agricultural University, Changchun 130118, China.

2 Institute of Crop Science, Chinese Academy of Agricultural Sciences (CAAS)/National Key Facility for Crop Gene Resources and Genetic Improvement, Key Laboratory of Biology and Genetic Improvement of Triticeae Crops, Ministry of Agriculture, Beijing 100081, China.

3 Soybean Research Institute, Jilin Academy of Agricultural Sciences/National Engineering Research Center for Soybean, Changchun 130033, China.

4 Crop Resources Institute of Heilongjiang Academy of Agricultural Sciences, Harbin 150000, China.

5 College of Modern Agriculture, Changchun Vocational Institute of Technology, Changchun 130118, China.

† These authors contributed equally to this work.

* Corresponding author: majian197916@jlau.edu.cn and xuzhaoshi@caas.cn

This PDF file includes:

Tables S1 to S3

Figures S1 to S5

Table S1. Details of the 83 soybean PP2A-B" genes, including gene ID, protein sequence length, predicted molecular weight (MW), predicted theoretical p*I*, and chromosomal location.

Gene ID	Number of amino acids	Molecular weight（Da)	Theoretical p*I*	Chromosome
GmPP2A-B"01	187	21452.45	4.66	1
GmPP2A-B"02	214	24265.7	5.22	1
GmPP2A-B"03	187	21203.16	7.65	1
GmPP2A-B"04	193	21997.38	4.98	2
GmPP2A-B"05	137	15497.5	4.2	2
GmPP2A-B"06	185	21121.94	6.75	2
GmPP2A-B"07	141	15896.77	4.62	2
GmPP2A-B"08	84	9321.3	4.32	2
GmPP2A-B"09	138	15732.94	4.43	2
GmPP2A-B"10	214	24327	5.66	3
GmPP2A-B"11	152	17000.16	4.32	3
GmPP2A-B"12	219	23931.74	4.6	3
GmPP2A-B"13	95	10929.21	9.66	4
GmPP2A-B"14	178	19719.58	4.65	4
GmPP2A-B"15	141	16041.27	4.44	4
GmPP2A-B"16	144	16585	4.79	4
GmPP2A-B"17	141	16053.26	4.39	4
GmPP2A-B"18	160	17402.74	4.12	4
GmPP2A-B"19	140	15524.92	4.5	4
GmPP2A-B"20	156	17937.29	4.52	4
GmPP2A-B"21	161	17579.06	4.35	5
GmPP2A-B"22	81	9167.32	4.72	5
GmPP2A-B"23	188	20432.67	4.28	5
GmPP2A-B"24	187	20833.43	4.29	6
GmPP2A-B"25	95	10917.36	9.99	6

GmPP2A-B"26	95	10862.2	9.96	6
GmPP2A-B"27	160	17443.94	4.33	6
GmPP2A-B"28	179	19459.2	4.26	7
GmPP2A-B"29	182	19958.66	4.63	7
GmPP2A-B"30	185	21204.93	6.84	7
GmPP2A-B"31	164	19025.43	4.64	7
GmPP2A-B"32	147	15884.69	4.55	7
GmPP2A-B"33	180	19704.47	4.32	8
GmPP2A-B"34	80	9236.43	4.66	8
GmPP2A-B"35	137	15281.13	4.44	8
GmPP2A-B"36	229	26116.6	4.68	8
GmPP2A-B"37	216	25189.81	5.26	8
GmPP2A-B"38	88	9629.29	4.67	9
GmPP2A-B"39	207	24061.12	4.44	9
GmPP2A-B"40	183	20521.13	4.48	9
GmPP2A-B"41	130	14290.16	4.88	10
GmPP2A-B"42	187	21048.87	7.69	11
GmPP2A-B"43	141	16134.34	4.32	11
GmPP2A-B"44	141	16132.31	4.32	11
GmPP2A-B"45	159	17783.04	4.8	11
GmPP2A-B"46	137	15491.55	5.07	11
GmPP2A-B"47	102	11171.12	4.22	11
GmPP2A-B"48	140	15387.9	4.49	12
GmPP2A-B"49	163	18112.33	4.6	12
GmPP2A-B"50	123	13951.66	4.51	12
GmPP2A-B"51	711	79666.77	5.44	12
GmPP2A-B"52	501	56051.34	6.07	12
GmPP2A-B"53	164	18842.23	4.24	13
GmPP2A-B"54	157	16944.76	4.05	13

GmPP2A-B"55	229	25582.18	4.75	13
GmPP2A-B"56	579	63564.08	5.36	13
GmPP2A-B"57	712	80314.76	5.57	13
GmPP2A-B"58	712	79963.09	5.43	13
GmPP2A-B"59	138	15753.99	4.4	14
GmPP2A-B"60	144	16276.86	4.32	14
GmPP2A-B"61	131	14085.03	5.42	14
GmPP2A-B"62	163	18548.91	4.24	14
GmPP2A-B"63	141	15892.7	4.62	14
GmPP2A-B"64	84	9336.36	4.42	14
GmPP2A-B"65	211	23934.34	4.9	15
GmPP2A-B"66	137	15189.08	4.43	15
GmPP2A-B"67	140	15965.98	4.49	16
GmPP2A-B"68	229	26224.89	4.7	16
GmPP2A-B"69	160	17610.93	4.23	16
GmPP2A-B"70	192	21941.26	4.96	16
GmPP2A-B"71	156	16730.7	4.45	17
GmPP2A-B"72	152	16828.73	4.63	17
GmPP2A-B"73	229	25837.42	4.63	17
GmPP2A-B"74	140	16018.6	4.63	17
GmPP2A-B"75	190	20993.62	4.79	17
GmPP2A-B"76	139	15621.61	4.98	18
GmPP2A-B"77	223	25418.73	4.68	18
GmPP2A-B"78	207	24087.11	4.51	18
GmPP2A-B"79	137	15508.61	4.25	18
GmPP2A-B"80	137	15468.51	4.27	18
GmPP2A-B"81	152	16957.97	4.25	19
GmPP2A-B"82	218	24040.91	4.69	19
GmPP2A-B"83	130	14434.42	5.2	20

Table S2. The primers used for the experiments.

For qRT-PCR

Name	Sequence (5'-3')
**GmActin-F**	GTTGAAAAGCCAGGGGACA
**GmtActin-R**	TCTTACCCCTTGAGCGTGG
**GmPP2A-B"12-F**	CGGTAACCAAACTCCGACCA
**GmPP2A-B"12-R**	AGAGACTCAACCGTGATGCC
**GmPP2A-B"33-F**	GTGTACCTGCAAGACTCGGA
**GmPP2A-B"33-R**	CAGGTTGAAGGCATCGTGGA
**GmPP2A-B"36-F**	GAAACTACATCAGGGGAGCGT
**GmPP2A-B"36-R**	AGCCTCGTCATTTTCCTCATCGT
**GmPP2A-B"46-F**	GCCTGAGGAGTTTAGCCACC
**GmPP2A-B"46-R**	CCTGCTTCACCTCCTCCAA
**GmPP2A-B"56-F**	GCATTGTTGGTGGCCTCTTC
**GmPP2A-B"56-R**	CCAGCTAGCAGTCCCATTCC
**GmPP2A-B"68-F**	CACCCAAAAAGGGATCAGTGC
**GmPP2A-B"68-R**	AAATGTGCGCTCCAGCTTTG
**GmPP2A-B"69-F**	TCTTTCGACCGTGATGGCAA
**GmPP2A-B"69-R**	CGTTGAAGCTAATGACGCCG
**GmPP2A-B"71-F**	GATCGGGGCAATGATGACAG
**GmPP2A-B"71-R**	TAGCGGGGGAACAATCAAGC
**GmPP2A-B"82-F**	CGGTAACCAAACTCCGACCA
**GmPP2A-B"82-R**	AGCCTTTCTACGGTGATGCC
**GmCAT1-F**	GAACAACTTCAAGCAGCCCG
**GmCAT1-R**	GCCTCGTGCTGAGATGAGAA
**GmCAT2-F**	CAGGCATATGGATGGCTTCG
**GmCAT2-R**	GGTGGCATGACTGTGGTTGG
**GmPOD1-F**	ACATTGGAGTGCTAACGGGA
**GmPOD1-R**	TGAGCTAACCATGCCATCTGA

**GmLEA15-F**	AGCACAACTACGAGACCAGC
**GmLEA15-R**	CCCATCATCCTCAGACGAGC
**GmERF115-F**	CACCTCACAAATCAGCACAGC
**GmERF115-R**	TGTTACTCCCACCACCCATGA

For cloning

**Name**	**Sequence (5'-3')**
**GmPP2A-B"71-F**	ATGTGCCCTTCCGGTCGCA
**GmPP2A-B"71-R**	TTAATTAGCGGGGGAACAATCAAG

Table S3 Soil moisture content (g)

Arabidopsis	**Normal**	**Drought**
**Replication 1**	26.83±1.2	7.96±0.5
**Replication 2**	27.52±0.9	8.23±0.6
**Replication 3**	27.24±1.2	8.15±0.6
**Replication 4**	27.99±1.0	8.99±0.4
**Replication 5**	27.94±1.1	7.88±0.5
**Average**	27.504	8.242

**Soybean**	**Normal**			**Drought**		
	GmPP2A-B'' *71*-RNAi	EV-control	*GmPP2A-B''* *71*-OE	*GmPP2A-B''* *71*-RNAi	EV-control	*GmPP2A-B''* *71*-OE
**Replication 1**	26.98±1.2	26.89±0.9	27.84±1.3	7.95±0.6	7.85±0.6	8.05±0.4
**Replication 2**	27.23±1.1	27.84±1.1	26.84±0.8	8.11±0.5	7.94±0.3	8.09±0.5
**Replication 3**	27.64±0.9	27.85±1.2	26.83±1.2	8.19±0.5	8.1±0.5	8.13±0.6
**Replication 4**	26.56±0.9	26.89±1.3	27.33±1.2	7.99±0.6	8.2±0.6	8.12±0.4
**Replication 5**	27.99±1.1	27.7±1.1	28.05±1.1	8.06±0.4	7.9±0.5	8.09±0.5
**Average**	27.28	27.434	27.378	8.06	7.998	8.096

Table S3. After two weeks of drought treatment, the soil water content of the control group and the experimental group.

Figure S1

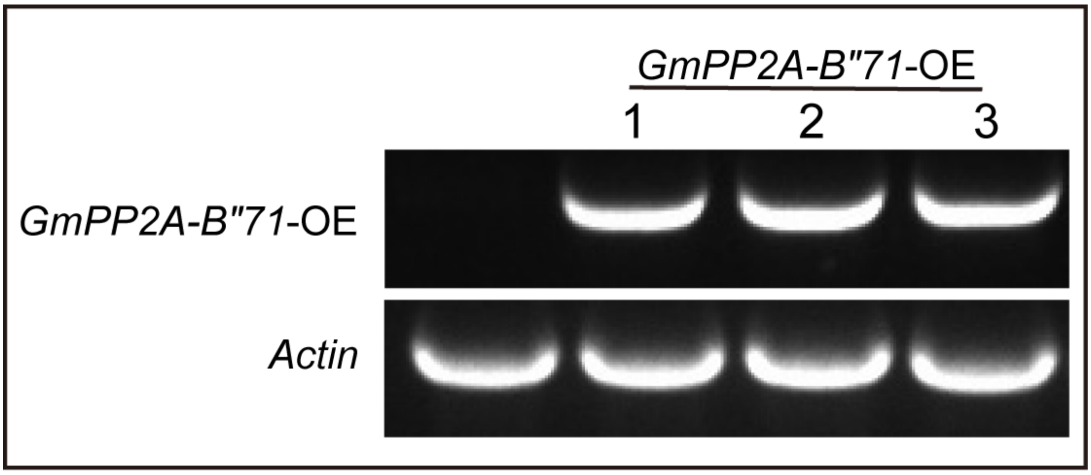

Figure S1. Identification analysis of three transgenic *Arabidopsis* lines. *Actin* as the reference gene.

Figure S2

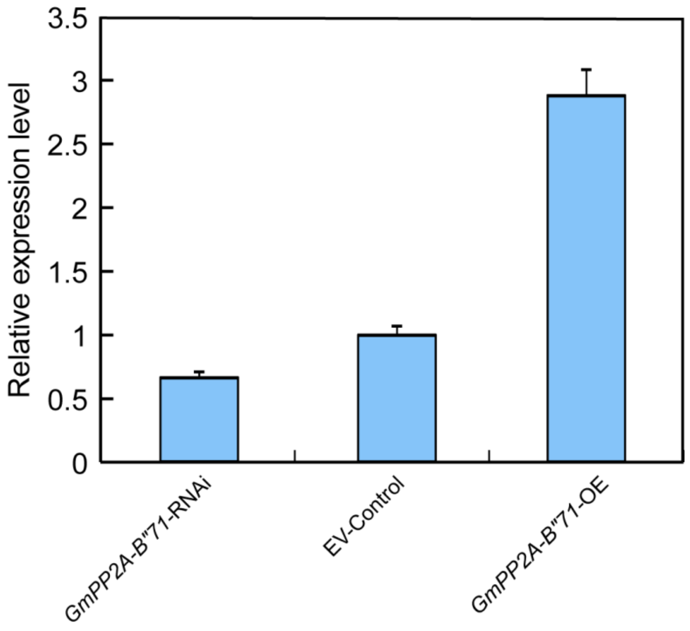

Figure S2. qRT-PCR analysis of *GmPP2A-B"71* expression levels in *GmPP2A-B"71*-RNAi, EV-Control, and *GmPP2A-B"71*-OE transgenic hairy roots. The data are shown as means of three biology repeats ± SD.

Figure S3

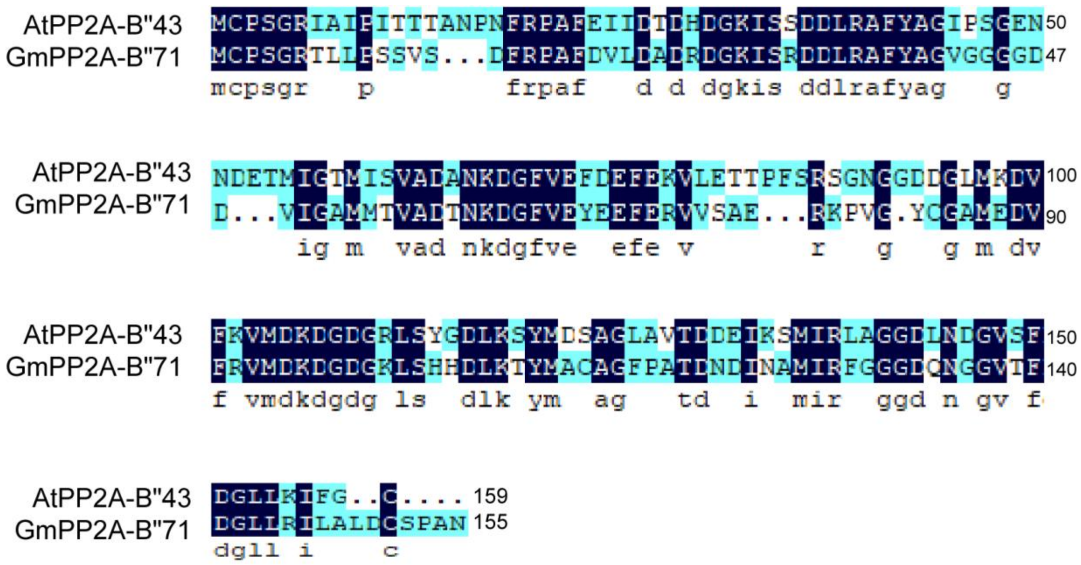

Figure S3. Alignment of AtPP2A-B"43 and GmPP2A-B"71 amino acid sequences.

Figure S4

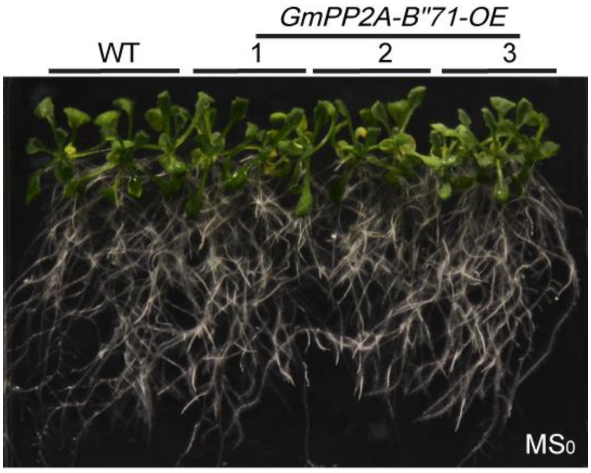

Figure S4. WT and three transgenic *Arabidopsis* lines grown on MS_0 medium.

Figure S5

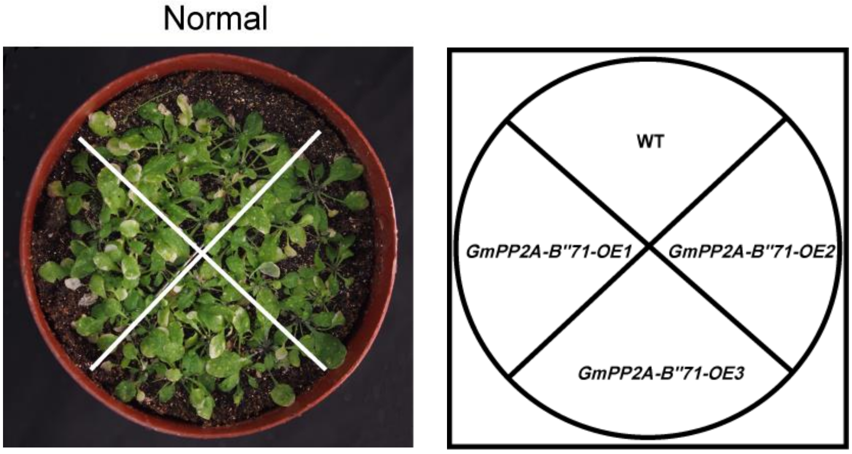

Figure S5.WT and three transgenic *Arabidopsis* lines grown under normal conditions
